# *Nocardioides*: “Specialists” for Hard-to-Degrade Pollutants in the Environment

**DOI:** 10.3390/molecules28217433

**Published:** 2023-11-05

**Authors:** Yecheng Ma, Jinxiu Wang, Yang Liu, Xinyue Wang, Binglin Zhang, Wei Zhang, Tuo Chen, Guangxiu Liu, Lingui Xue, Xiaowen Cui

**Affiliations:** 1 College of Biotechnology and Pharmaceutical Engineering, Lanzhou Jiaotong University, Lanzhou 730070, China; 2 Key Laboratory of Extreme Environmental Microbial Resources and Engineering, Lanzhou 730000, China; 3 Key Laboratory of Desert and Desertification, Northwest Institute of Eco-Environment and Resources, Chinese Academy of Sciences, Lanzhou 730000, China; 4 State Key Laboratory of Cryospheric Sciences, Northwest Institute of Eco-Environment and Resources, Chinese Academy of Sciences, Lanzhou 730000, China; 5 College of Geography and Environment Science, Northwest Normal University, Lanzhou 730070, China

**Keywords:** biodegradation, environmental remediation, *Nocardioides*, organic contaminants

## Abstract

*Nocardioides*, a genus belonging to *Actinomycetes*, can endure various low-nutrient conditions. It can degrade pollutants using multiple organic materials such as carbon and nitrogen sources. The characteristics and applications of *Nocardioides* are described in detail in this review, with emphasis on the degradation of several hard-to-degrade pollutants by using *Nocardioides*, including aromatic compounds, hydrocarbons, haloalkanes, nitrogen heterocycles, and polymeric polyesters. *Nocardioides* has unique advantages when it comes to hard-to-degrade pollutants. Compared to other strains, *Nocardioides* has a significantly higher degradation rate and requires less time to break down substances. This review can be a theoretical basis for developing *Nocardioides* as a microbial agent with significant commercial and application potential.

## 1. Introduction

Several pollutants, including heavy metals, petroleum, and organic pollutants such as aromatic compounds, etc., are currently polluting the environment [1]. These pollutants are highly toxic, stable, and challenging to degrade [2] and have potent carcinogenicity [3]. They pose a severe threat to the environment and public health [4]. Physical transfer adsorption, chemical precipitation oxidation, biological precipitation dissolution, etc., are used to treat common pollutants [5]. Extraction, adsorption, and membrane separation are the often-used physical remediation methods. Nonetheless, they are ineffective, expensive, and prone to secondary pollution [6]. Chemical precipitation, electrolytic oxidation and reduction, and photochemical remediation are examples of chemical remediation methods [7]. Applying chelated precipitation and chemical modifiers makes it easy for the soil’s environmental structure to become damaged and produce secondary pollution [8]. The microorganisms that make up bioremediation technology are used to adsorb, degrade, or transform environmental pollutants into other harmless substances [9]. According to the chosen mechanism, there are now three standard microbial remediation techniques: (1) biosorption and enrichment [10]; (2) biodegradation [11]; and (3) biological precipitation and dissolution [12]. Adsorbed ions in microbial cells can be categorized into three groups based on how they are distributed: through internal, external, or surface adsorption [13]. Biosorption is frequently used to treat heavy metals. For example, *Bacillus* NMTD17 can reach cadmium (Cd^2+^) biosorption equilibrium after 60 min, and its maximum Cd^2+^ adsorption capacity is 40 mg/L [14]. Biodegradation uses its metabolic capacity, including membrane transport, enzyme degradation, and carbohydrate metabolism [15]. For example, *Clostridium* sp. can metabolize trichloroethylene (TCE) into the less toxic dichloride [16]. Similarly, the fungi represented by *Candida tropicalis* can degrade phenol using their pheA-encoded phenol hydroxylase [17] and catechol 1-dioxygenase [18] encoded by *catA*. Organic acids secreted by organisms help dissolve and precipitate pollutants through biological precipitation and dissolution. For example, *Acidithiobacillus* can produce sulfuric acid, and it can convert the insoluble metal in soil into soluble sulfates by acidifying the soil [19]. Microbial remediation technology outperforms other remediation technologies in terms of efficiency and cost. For example, petroleum hydrocarbons’ microbial degradation costs roughly 50–70% less than chemical and physical methods [20]. Second, there is no secondary pollution, and the conditions for microbial degradation are milder [21]. Therefore, bioremediation technologies represented by microorganisms should be given priority when solving the problems caused by environmental pollution.

**Figure 1:**
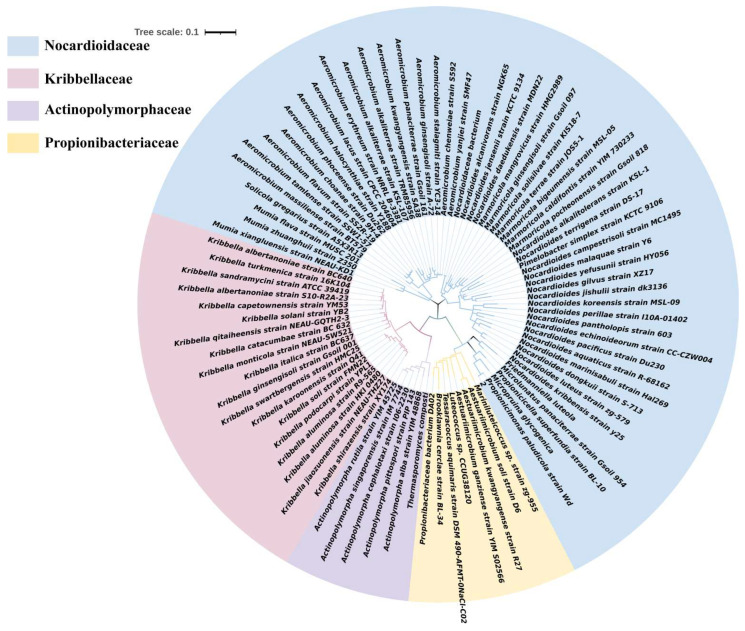
A phylogenetic tree of *Nocardioidaceae* belonging to the order *Propionibacteriales* of *Actinobacteria*.

*Nocardioidaceae* is a family within the order *Propionibacteriales*, as shown in Figure 1. There are 158 effective species of *Nocardioides*, a type of rare *Actinomycetes* with a similar evolutionary relationship and morphology [22]. Strains other than *Streptomyces* are frequently classified as the rare *Actinomycetes* [23]. *Nocardioides* can use a variety of organic substances as carbon sources, including petroleum hydrocarbons, aromatic compounds, and nitrogen heterocyclic compounds [24]. As described in Table 1, *Nocardioides* can degrade a variety of pollutants. They can be divided into four categories: aromatic compounds, hydrocarbon and haloalkane, nitrogen heterocyclic, and polyester pollutants, such as nitrophenol, cotinine, ritalinic, polylactic acid, etc. This signals that *Nocardioides* has a wide range of prospects for pollutants. *Nocardioides* sp. KP7 [25] has the benzene-ring degradation genes *phdA*, *phdB*, *phdC*, and *phdD*. They can code for the enzymes involved in degradation, which can degrade to phthalates using phenanthrene as their carbon source. This discovery was made as early as 1999. *Nocardioides*’ degradation currently affects several fields, including medicine [26,27], industry [28], materials [29], etc. The common ones include 2,4-dinitroanisole [30], dibenzofuran [27], nitrophenol [21], and ibuprofen [31]. Consider the following example: at an initial concentration of 1.5 mg/L, strain CBZ_1T eliminated 70% of ibuprofen in 7 days [32].

In addition to degrading organic pollutants, some strains of *Nocardioides* are known to be effective at carrying out steroid biodegradation and biotransformation. Steroids are biomolecules in higher organisms that perform basic physiological functions [44]. Steroids are widely used in different fields of medicine. At the same time, steroids are emerging contaminants (ECs) [45]. Steroids are a class of endocrine disruptors that, at very low levels, can lead to some adverse effects such as sex hormone imbalance, decreased reproductive ability, and cancer in organisms, so the problem of steroid hormone pollution in the environment has attracted widespread attention from researchers [46]. *Nocardioides simplex* VKM Ac-2033D has high 3-ketosteroid 1(2)-dehydrogenase activity toward a wide range of steroids, such as androstenedione, progesterone, hydrocortisone, 6α-methylhydrocortisone, cortexolone, and 21-acetyl-cortexolone [47]. *N. simplex* VKM Ac-2033D can convert 92% of hydrocortisone (5 g/L) into prednisolone in 2 h [47]. *N. simplex* VKM Ac-2033D can also convert pregna-4,9(11)-diene-17α and 21-diol-3,20-dione acetates [48]. By conducting omics studies on the bacteria, *N. simplex* VKM Ac-2033D was found to possess genes related to the sterol uptake system and aliphatic side-chain degradation at C17 and A/B- and C/D-ring degradation systems [49]. It can introduce a ∆1-double bond in various 1(2)-saturated 3-ketosteroids and perform the conversion of 3β-hydroxy-5-ene steroids to 3-oxo-4-ene steroids, the hydrolysis of acetylated steroids, and the reduction of carbonyl groups at C-17 and C-20 of androstanes and pregnanes, respectively [49]. Meanwhile, *N. simplex* VKM Ac-2033D can completely degrade cholesterol and lithocholate at an initial concentration of 1 g/L in 72 h. The strain is able to grow on cholesterol as well as lithocholate as the sole carbon and energy sources [50]. Phytosterol can also be completely degraded by *N. simplex* VKM Ac-2033D at an initial concentration of 1 g/L in 120 h [51].

This review summarizes the fundamental traits of *Nocardioides* before focusing on the types of pollutants that *Nocardioides* can degrade. Simultaneously, the ability of *Nocardioides* to degrade pollutants is introduced. This review provides the specific degradation pathways for representative pollutants. Researchers require such information in order to develop and apply microbial degradation methods for environmental remediation.

## 2. *Nocardioides*

**Figure 2:**
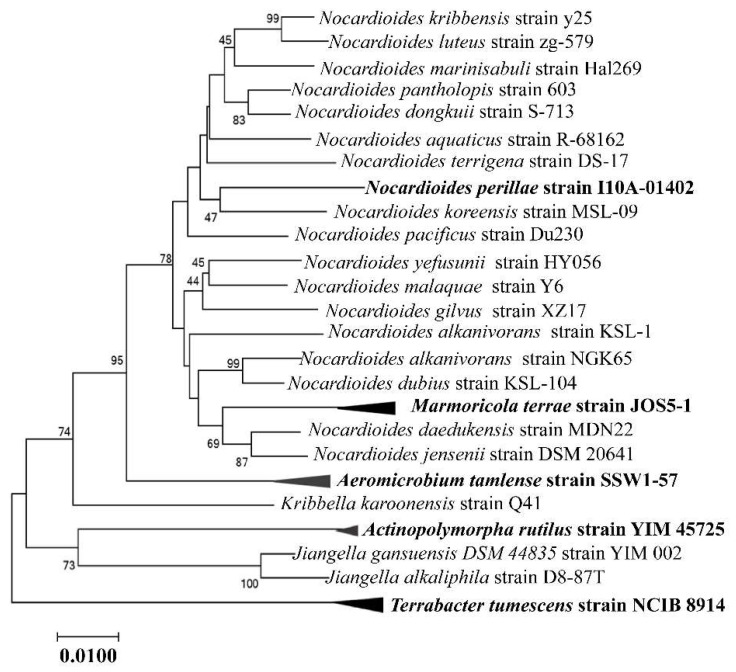
Phylogenetic dendrogram obtained via neighbor-joining using the 16s rRNA gene sequences of *Nocardioides* and related strains. (The numbers on the branch nodes are bootstrap values.)

*Nocardioides* was first known as *Nocardia*. It differs from regular *Actinomycetes* in that it has irregularly branching aerial hyphae, and the transverse septum breaks into rods or globules [52]. In 1976, Prauser H [53] isolated seventeen strains of *Actinomycetes* from soil, each with unique taxonomic traits, and based on their distribution source, morphology, physiological and biochemical characteristics, etc., classified them as a new genus of *Actinomycetes*. *Nocardioides albus* served as the type species for the newly recognized genus [53]. The LL-2,6-diaminopimelic acids (LL-DAP) and lack of branching acid distinguish *Nocardioides* from *Nocardia*. In 1985, Nesterenko et al. established *Nocardioidaceae* [22]. According to phylogeny, the three most recent genera are *Nocardioides*, *Marmoricola*, and *Aeromicrobium*, as shown in Figure 2.

**Figure 3:**
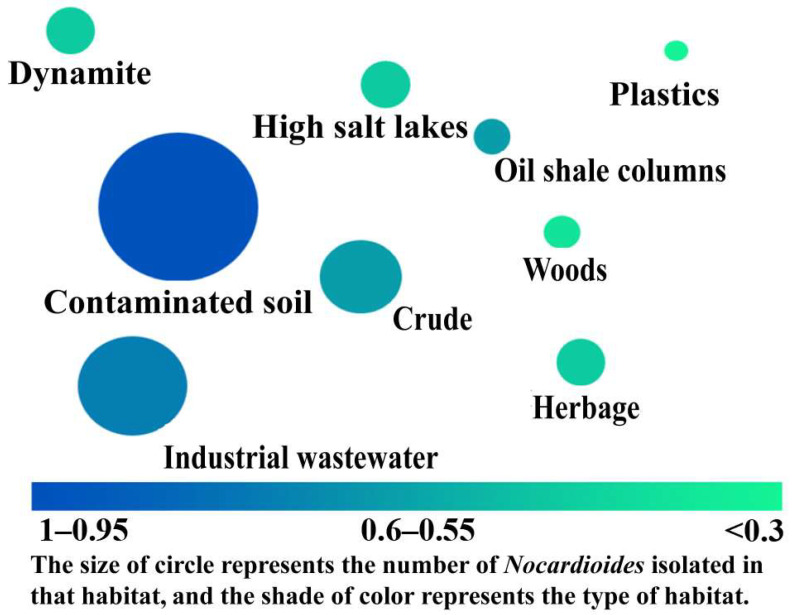
Types of *Nocardioides* habitats and the *Nocardioides*’ distribution in different habitats.

*Nocardioides* bacteria are aerobic, Gram-positive, and globular or irregularly rod-shaped [54]. The majority of *Nocardioides*’ aerial hyphae have sparse or irregular branches and measure about 1.0 μm in length [53]. Only a few *Nocardioides* (*Nocardioides simplex*, *Nocardioides jensenii*, *Nocardioides plantarum*, *Nocardioides pyridinolyticus*, *Nocardioides nitrophenolicus*, and *Nocardioides aquaticus*) lack aerial hyphae. As the culture time increases, the cell morphology gradually changes from rod-shaped to cocciform [53]. The colony has a smooth and glossy, round, neatly defined edge and a color that ranges from slightly white to light yellow. The best growth temperature is 28–30 °C, and the best growth pH is 7–8. Most organisms require salt but are not halophilic (often isolated from marine and marine-related environments). These organisms typically need 0.5–6% NaCl to thrive [22]. As demonstrated in Figure 3, *Nocardioides* can also grow and reproduce using various organic chemicals in different contaminated habitats, such as industrial wastewater, contaminated soil, crude oil, etc. Figure 3 summarizes the main habitat types of *Nocardioides* and the *Nocardioides*’ distribution in different habitats. The size of the circle represents the number of *Nocardioides* isolated in that habitat, and the shade of color represents the type of habitat. Figure 3 shows that there are eight types of *Nocardioides* habitats and the main habitats of *Nocardioides* are contaminated soil and industrial wastewater. Industrial wastewater is the second most common source of isolation for *Nocardioides*. This signals that *Nocardioides* has great potential for degrading pollutants.

**Figure 4:**
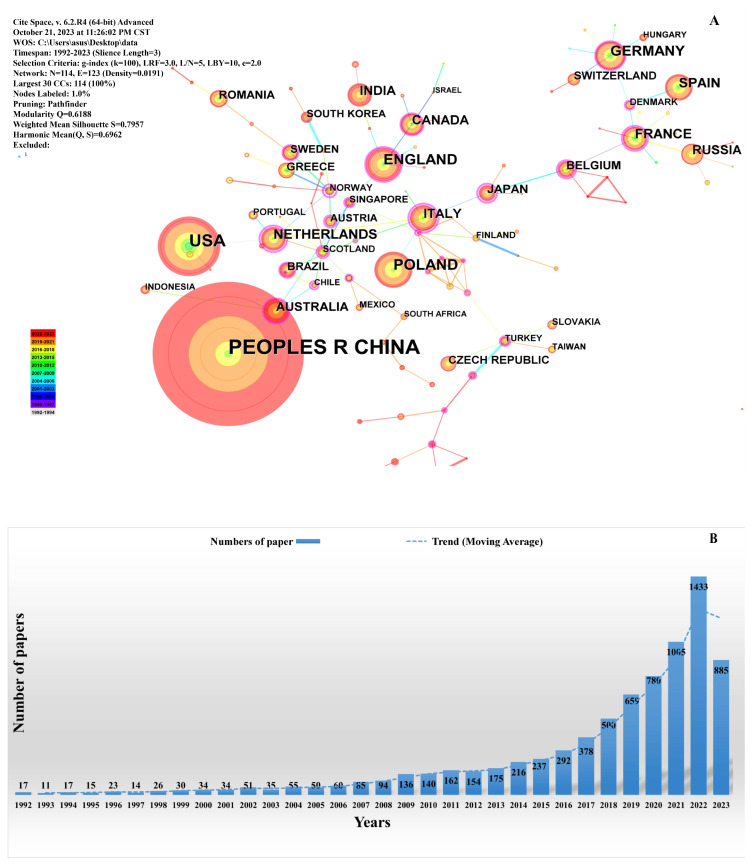
Bibliometric statistics of *Nocardioides*. (**A**) *Nocardioides* research countries and (**B**) number of *Nocardioides* publications.

**Figure 5:**
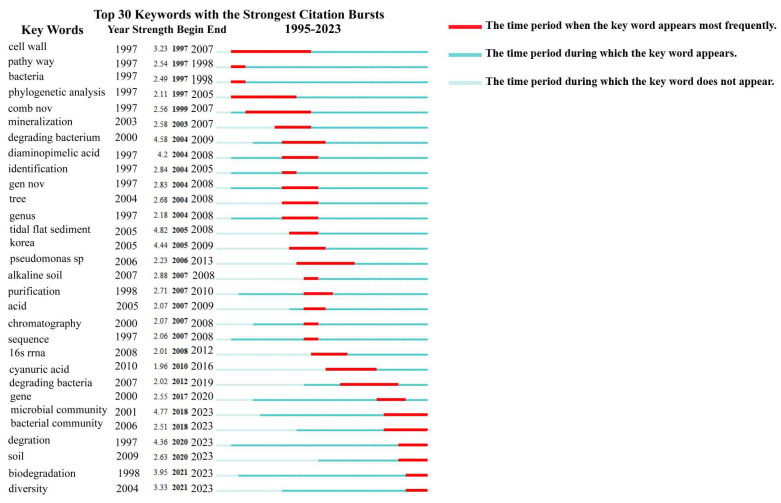
Keyword map of *Nocardioides* research highlights.

Through examining the research statistics on *Nocardioides* from the past 30 years, it was found that research on *Nocardioides* in the past 5 years has gradually increased. As shown in Figure 4A, the countries where more research has been conducted are China, the United States, Poland, Germany, Russia, etc. The number of *Nocardioides* publications has also increased dramatically, as shown in Figure 4B. As shown in Figure 5, current *Nocardioides* research focuses on pollutant degradation. Researchers discovered the nitrophenol-degrading *Nocardioides nitrophenolicus* sp. NSP41T in 1999. *Nocardioides carbamazepini* sp. nov. [26] and *Nocardioides* sp. [18], which can degrade ibuprofen and nitrophenol, were isolated by researchers in 2022. Over the last 50 years, research on *Nocardioides* has continuously grown, and mining for new species and determining their ability to degrade environmental pollutants have both gained popularity. *Nocardioides* has also gradually demonstrated the ability to degrade pollutants. This suggests that there is more to explore regarding *Nocardioides* than other *Actinomycetes* and that it is possible to discover new species and application values.

## 3. Applications of *Nocardioides*

With the gradual discovery of new species of *Nocardioides*, *Nocardioides* exhibit good pollutant degrading skills. Notably, some refractory pollutants, such as ritalinic, atrazine, and polylactic acid [40,55], are closely related to different aspects of life, involving medicine, industry, etc. It is important to summarize the type and ability of *Nocardioides* to degrade these pollutants. This provides more possibilities for microorganisms to repair the environment and protect its ecology. In this review, pollutants are divided into five categories according to their chemical structure: hydrocarbons, halogenated alkanes, aromatic compounds, nitrogen heterocyclic pollutants, and polyester pollutants. A detailed summary of the types and abilities of *Nocardioides* to degrade pollutants is presented.

### 3.1. The Degradation of Hydrocarbon and Haloalkane Pollutants

Common hydrocarbon pollutants include crude oil [16], butane [56], etc. One of the world’s most significant energy sources is crude oil, and as industrialization advances exponentially, demand is growing [57]. However, oil spillage during extraction, shipping, and refinement can severely pollute the land [57]. The chemical wastewater released by the chemical printing and dyeing industries also contains a variety of petroleum hydrocarbon pollutants, which harm the soil’s ecological ecosystem and contaminate the water body [58,59]. Petroleum hydrocarbons can also lower crop yield because they accumulate in plants, interfere with their normal physiological processes, and inhibit plant photosynthesis [60]. These pollutants risk human health and can harm the respiratory system by entering the human body through various pathways and accumulating in organisms [61].

Many different types of microorganisms in nature can degrade petroleum pollutants, including *Pseudomonas* spp. [62], *Bacillus* sp. [61], *Nocardioides* sp. [57], etc. Alkanes are a carbon source that *Nocardioides* can use [56]. For instance, Hamamura et al. [44] discovered that *Nocardioides* sp. strain CF8 was found to have butane monooxygenase [62], which may use butane and a variety of alkanes as carbon sources [63]. The *Nocardioides luteus* strain BAFB [63] degrades the C11 alkanes in jet fuel JP-7 by using them as a carbon source in long-chain alkanes. *Nocardioides oleivorans* sp. and *Nocardioides* sp. were also isolated from crude oil samples of oil fields by Schippers et al. and Roy et al. Both may utilize crude oil as a carbon source, while *Nocardioides oleivorans* sp. can adapt to the condition of a maximum of 50 mg/mL of crude oil, and it can degrade 40% of 50 mg/mL crude oil as its carbon source.

Halogenated hydrocarbons are byproducts produced when halogen groups replace hydrogen atoms in hydrocarbon molecules. The presence of halogen atoms makes the molecule more poisonous [64]. Vinyl chloride (VC), an extremely dangerous and carcinogenic halogenated hydrocarbon, is widely found in groundwater and soil [65]. It was included in the 2017 list of class I carcinogens due to its widespread use in the polymer chemical industry [66]. VC is a severe hazard to the environment and people’s health [67]. *Dehalococcoides* spp. [68], *Nocardioides* sp. [69], etc., are the common VC-degrading bacteria. According to Mattes et al., *Nocardioides* sp. strain JS614 may use VC as a carbon source, and the *etnE* gene encodes epoxy alkyl coenzyme M transferase, which breaks down VC [70]. Additionally, Wilson et al. confirmed that *Nocardioides* sp. may use VC as a carbon source [71]. *Nocardioides* sp. is primarily concerned with the degradation of crude oil and the utilization of VC. *Nocardioides* can be observed to have various degradation types for hydrocarbon and haloalkane pollutants.

### 3.2. The Degradation of Aromatic Compounds

Aromatic compounds with stable chemical structures, typical carcinogenicity, and mutagenicity have been discovered in various natural habitats, such as soil and water [72]. In addition to significantly inhibiting microorganisms, these toxic compounds threaten human health and the natural environment, and preventing this is the primary goal of pollution control [73]. Additionally, the quantity of benzene rings in aromatic compounds is positively correlated with the difficulty of carrying out the environmental degradation of aromatic compounds and their toxicity [65]. In a study, it was found that their volatility decreased as the number of benzene rings increased, the solubility in fat increased, and the difficulty of environmental degradation increased. Due to their high level of carcinogenicity, teratogenicity, mutagenicity, and ecological toxicity [69], aromatic compounds—which are typically present in water, soil, and sediments [68]—pose a severe risk to human health and the environment [74]. *Nocardioides* has been found to degrade aromatic compounds such as 2-dinitroanisole, ibuprofen, dibenzofuran, and nitrophenol.

**Figure 6:**
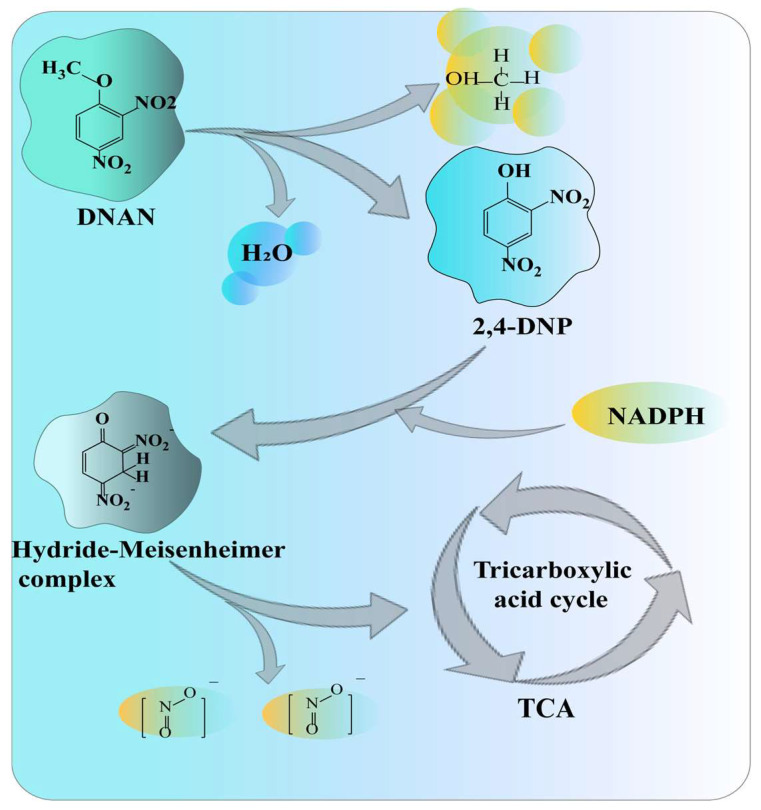
Proposed pathway for *Nocardioides* sp. JS1661 to degrade DNAN [64].

2,4-dinitrophenol (DNAN) is a typical aromatic compound. It gradually substitutes trinitrotoluene (TNT) as a low-sensitivity explosive [29]. In addition to creating significant acute cytotoxicity during methanogenesis and nitrification, DNAN can also cause damage to algae, microorganisms, and plants. Karthikeyan et al. isolated a *Nocardioides* sp. JS1661 strain and determined that it could use DNAN as its only carbon source to degrade DNAN and release nitrite through the 2,4-dinitrophenol (DNP) pathway [29]. Figure 6 illustrates the degradation pathway. *N.* sp. JS1661 can adapt to the condition of a maximum of 150 mg/mL of DNAN. Additionally, within 45 h, *N.* sp. JS1661 can degrade 150 mg/L of DNAN. *Rhodococcus erythropolis* strain HL 24-1 can degrade 92 mg/L of DNAN. Its degradability is nearly twice that of *R. erythropolis* strain HL 24-1 [75]. The oxygen demethylation of DNAN is the first step in creating DNP and methanol [76]. The cleavage of the ether bond to form DNP, the formation of the hydride–Meisenheimer complex from DNP, and the release of nitrite are all processes catalyzed by DNAN demethylase. A study indicated that DNAN has little to no accumulation, nitrite has an almost stoichiometric release, and DNAN can be completely degraded within 20–50 h [30]. Microbial degradation is becoming more significant due to the increased use of DNAN. The degradation of polycyclic aromatic hydrocarbons (PAHs) by *Nocardioides* mainly involves an aerobic pathway, which is carried out by means of the hydroxylation of double oxygenation, dehydrogenation, and ring-opening double oxygenation [77]. Ring-hydroxylating oxygenase binds oxygen atoms to PAHs to produce cis-dihydrodiol, which continues to be metabolized and degraded by dehydrogenation and ring-opening steps. Unlike other bacteria, *Nocardioides* also has a cytochrome P450 monooxygenase pathway [78]. The enzyme also converts polycyclic aromatic hydrocarbons (PAHs) to cis-dihydrodiol, dehydrogenates them, converts them to diols, and then epoxides them to form intermediates in the tricarboxylic acid cycle, which is used in cell synthesis or catabolism. Examples of p-nitrophenol-degrading bacteria isolated from industrial wastewater include *Nocardioides* sp. KP7 [28], *Nocardioides nitrophenolicus* sp. NSP41T [79], and *Nocardioides simplex* FJ2-1A [80]. With the help of the two enzymes coenzyme F420 and ring-hydroxylating oxygenase, *N. simplex* FJ2-1A may mineralize and use TNT and DNP [80]. The 2,4,6-trinitrophenol requires coenzyme F420 to form a picric acid hydride σ-complex, which combines with DNAN to create a dihydrocomplex [30].

Ibuprofen is also a benzene-ring compound. It is a drug widely used as an antipyretic, pain reliever, etc. [28]. Ibuprofen contamination has been discovered in finished drinking water, surface and groundwater, and pollution from other medications and personal care products. Municipal and industrial wastewater effluents are the main entry points for ibuprofen into the environment [32]. Increases in ibuprofen use and drug residues eventually cause ecotoxicity [81]. The most prevalent bacteria that degrade ibuprofen include *Sphingomonas* sp., *Bacillus* sp., *Nocardioides* sp., etc. Carballa et al. found that at an initial concentration of 1.5 mg/L, in one week, ibuprofen’s biological oxidative removal rate was >70% in *Nocardioides*. Nevertheless, the metabolic byproducts (hydroxyibuprofen and carboxyl ibuprofen) produced by specific strains during oxidation have toxicological effects comparable to those of ibuprofen in the aquatic environment [28]. Tibor et al. isolated a strain of *Nocardioides carbamazepini* sp. nov. from ibuprofen-contaminated water. *Nocardioides* degrades ibuprofen when glucose and ibuprofen are used as co-substrates. The bacteria can degrade 70% of 1 mg/L ibuprofen within seven weeks.

Dibenzofuran (DBF) is a model compound for studying aromatic compounds’ degradation processes and polychlorinated dibenzofurans [82]. DBF is a hazardous, hard-to-degrade benzene-ring pollutant that can last in the environment for a long time [83]. It is frequently used in medicine, disinfectants, preservatives, dyes, etc. The most prevalent bacteria that can degrade DBF include *Burkholderia xenovorans* strain LB400T [84], *Sphingomonas* sp. RW1 [85], *Pseudomonas resinovorans* strain CA10 [86], *Rhodococcus* sp. strain YK2 [87], etc. Aerobic degradation is the primary form of the biodegradation of DBF by microorganisms [88]. According to some studies, DBF is degraded by a ring-opening reaction involving the action of a biphenyl-degrading enzyme; it is hydroxylated by a dioxygenase and undergoes additional ring-opening reactions to 2,2,3-trihydroxy biphenyl, oxygenation to form 2,4-hexadienoic acid and different formations of salicylic acid and dihydroxybenzoic acid, and then into the tricarboxylic acid cycle to achieve complete transformation [89]. Previously, Kubota et al. [26] isolated DBF-degrading bacteria from soils and sediments contaminated with various amounts of DBF and discovered that *Nocardioides aromaticivorans*, a member of the Gram-positive *Actinomycetes*, was the most prevalent among the culturable DBF-degrading bacteria. *Nocardioides* has strong potential for dibenzofuran degradation. Simultaneously, *N. aromaticivorans* can adapt to the condition of a maximum of 33mg/L of DBF. It can also completely degrade 33 mg/L of DBF [26] within 96 h at pH 7 and 30 °C. *Pseudomonas* sp. strain C3211 was found to completely degrade 0.585 mg/L of DBF within 67 h [90], meaning that the degradation rate was over fifty-six times higher.

*Nocardioides* can also use several other aromatic pollutants as carbon sources, as shown in Table 1. *Nocardioides* outperforms different strains in its ability to degrade phenol pollutants by offering more types of degradation and superior degradability.

### 3.3. The Degradation of Nitrogen Neterocyclic Pollutants

Heterocyclic compounds with nitrogen can also serve as carbon sources for *Nocardioides*. Pyrrole, indole, pyridine, quinoline, isoquinoline, and their derivatives are examples of common nitrogen heterocyclic compounds [88]. They are present in industrial wastewater, such as pesticide, coking, dye, pharmaceutical, and dye wastewater [10]. Nitrogen heterocyclic pollutants have lower biodegradability and face more difficulty in disrupting metabolic processes than polycyclic aromatic hydrocarbons [91]. They seriously impair the environment and people’s health and are carcinogenic, teratogenic, and mutagenic [92]. In one study, a Korean researcher extracted a new strain of *Nocardioides pyridinolyticus* sp. nov. which can use pyridine as a carbon source [79]. In 2018, Professor Qiu isolated the *Nocardioides* sp. strain JQ2195 [27] from contaminated wastewater near urban areas. The strain can adapt to the condition of a maximum of 500mg/mL of cotinine. It can also degrade 500 mg/L cotinine in 32 h using pyridine cotinine as the only carbon and nitrogen source. During the degradation process, 50% of the cotinine was converted into 6-hydroxy-cotinine and 6-hydroxy-3-succinylpyridine (HSP) intermediates [55].

Methyl phenylacetate is a drug prescribed for the treatment of deficiency hyperactivity disorder among other promotional drugs [37]. Water pollution can result from the presence of ritalinic acid (RA), the primary metabolite of methylphenidate. As a biomarker used to identify the presence of methylphenidate in sewage epidemiology, RA has been proposed [93]. *Arthrobacter* sp. strain MW1 Marta, *Phycicoccus* sp. strain MW4, *Nocardioides* sp. [93], etc., degrade RA. *Nocardioides* sp. strain MW5 [93] 2020, which can alter the N heterocyclic site of RA using RA as the only source of nitrogen and carbon, was also discovered by Woźniak-Karczewska et al. in 2020. Meanwhile, it was found that when RA was used, the bacteria could adapt to the condition of a maximum of 1 g/L RA. Additionally, the bacteria could completely degrade 1 g/L of RA in 4 h.

**Figure 7:**
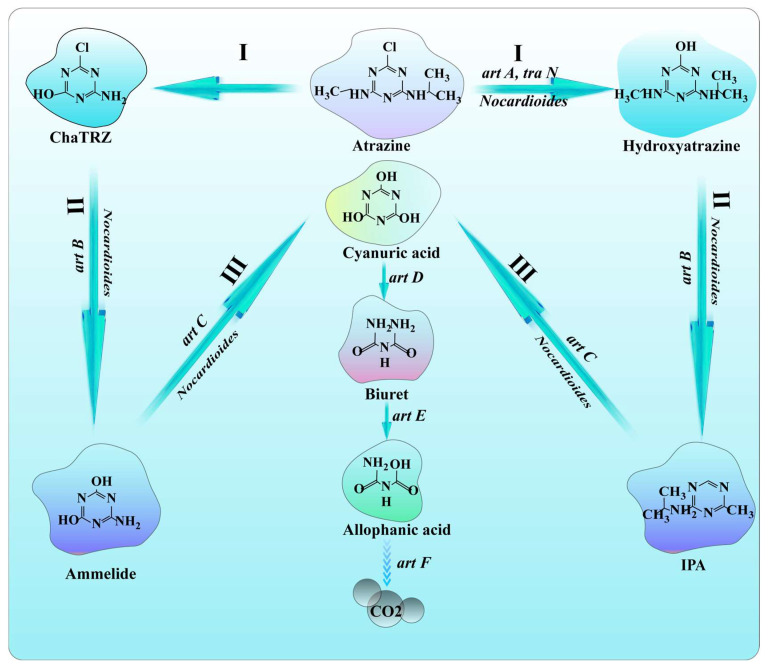
Proposed pathway and degrading genes of atrazine biodegradation by *Nocardioides*.

Triazines, such as triazine herbicides, are six-membered nitrogen heterocyclic molecules frequently used as insecticides [94]. Triazine herbicides were initially made available in China in the early 1980s. As their use has grown due to their high toxicity and endurance, they have not only affected the development of subsequent crops but also been found to be carcinogenic and harmful to human health [95]. According to some studies, *Nocardioides* sp. strain C190 could use atrazine as a carbon source [96]. Koji Satsuma discovered that *N*. strain DN36 could adapt to the condition of a maximum of 0.95mg/L of atrazine. It could completely degrade 0.95 mg/L of atrazine (triazine herbicides) in a week [38]. Dechlorination, dealkylation, hydroxylation, and ring cracking are some examples of specific degradation processes. The degradation genes of triazine herbicides include *atzA*, *atzB*, *atzC*, *atzD*, *atzE*, *atzF*, and *trzN* [97]. The function of the *trzN* gene is similar to that of *atzA*, which regulates dechlorination (step I) and then produces 2-amino-1 pyrrolidone under the control of the *atzB* gene (step II), followed by ammonia hydroxylation to cyanuric acid under the control of the *atzC* gene (step III). Then, *atzD* regulates the formation of cyanuric acid into biuret (step IV) and *atzE* regulates the removal of one amino group to isopropanoic acid (step V). *atzF* then generates carbon dioxide (step VI), as shown in Figure 7.

In addition, Takagi et al. isolated a strain of *Nocardioides* and discovered that the strain could adapt to the condition of a maximum of 5.04 g/L of melamine, and it was found to be able to degrade 5.04 g/L melamine (a nitrogen heterocyclic pollutant) [39] entirely in 20 d. Its ability to degrade melamine is nearly 50 times that of *Micrococcus* sp. strain MF-1 (100% degradation of 100 mg/L melamine) [98]. *Nocardioides* can degrade Ritalin, triazine herbicides, and melamine, and it has a variety of degradation pathways for insoluble nitrogen heterocyclic contaminants.

### 3.4. The Degradation of Polyester Pollutants

*Nocardioides* can degrade high-molecular-weight compounds such as biodegradable plastics: polyhydroxyalkanoates, polycaprolactone (PCL), poly (3-hydroxybutyrate) [P(3HB)], polylactic acid (PLA), etc. [99]. According to estimates, 300 million tons of plastic waste are produced annually worldwide, 79% of which is disposed of in landfills or released into the environment [100]. Biodegradation, in conjunction with plastics that degrade through microbial action, has gradually become one of the solutions to this problem [24]. Currently, *Marinobacter* sp., *Pseudomonas. stutzeri*, *Shewanella* sp., *Nocardioides* sp., etc., are the microorganisms known to degrade plastics [24]. Mitzscherling et al. isolated *Nocardioides alcanivorans* sp. from an environment polluted by plastics and *N. alcanivorans* NGK65T [101], which can use biodegradable plastics as a carbon source. Some scholars in Japan isolated a strain of *Nocardioides marinisabuli* OK12 from marine plastic waste which can use Poly-3-hydroxybutyrate (P(3HB)) as its only carbon source. The strain forms a biofilm on the surface of P(3HB). *Shewanella* sp. degraded P(3HB) at a rate of 47 μg/cm^2^/day, whereas strain OK12 degraded it at 318 ± 75 μg/cm^2^/day [41]. The degradation rate was found to be over seven times higher. Additionally, Mistry et al. constructed a combined bacterial agent containing *Nocardioides zeae* EA12, *Stentrophomonas pavanii* EA33, *Gordonia desulfuricans* EA63, and *Chitinophaga jiangningensis* EA02 that can completely degrade high-molecular-weight PLA film within 35 d [40].

*Nocardioides* combined with other microorganisms can completely degrade PLA, and P(3HB) impairs plastic significantly faster than different plastic-degrading strains. Several plastic pollution contaminants can be used to isolate *Shewanella* sp. and a novel species of *Nocardioides*. *Nocardioides* has excellent potential for degrading plastics, as has been demonstrated. In the future, *Nocardioides* is expected to become the “star” of biodegradable plastics.

## 4. Conclusions

Natural habitats contain *Nocardioides*, a rare form of *Actinomycetes*. Members of *Nocardioides* have been discovered and used due to the pure culture’s widespread use and the polyphasic classification of microorganisms. In most cases, *Nocardioides* is an aerobic Gram-positive bacteria with broken transverse septa that form rods or globules and uneven aerial hyphae [52]. LL-DAP and the absence of branching acid distinguish *Nocardioides* from *Nocardia* [22]. Presently, 158 effective *Nocardioides* species are known [22]. *Nocardioides* started relatively late when compared to other conventional *Actinomycetes*. The abundance of undiscovered new species is one of *Nocardioides*’ advantages. This undiscovered activity fills a gap in the connection of *Nocardioides* bacterial cultures and suggests we can investigate further undiscovered biological functions.

Additionally, preliminary findings from researchers suggest that it can degrade various pollutants, particularly refractory pollutants, including aromatic compounds, hydrocarbon and haloalkane pollutants, nitrogen heterocyclic pollutants, polymer polyester compounds, etc. Table 2 compares and summarizes the degradation by *Nocardioides* and other strains of pollutants. *Nocardioides* outperformed other strains in terms of their ability to degrade poly-3-hydroxybutyrate, dibenzofuran, 2,4-dinitrophenol, pyridine, and melamine, which can all be completely degraded. *N. marinisabuli* strain OK12 has a degradative capacity for poly-3-hydroxybutyrate that is about 7 times more than *Shewanella* sp., nearly 10 times as much as *Rhizobium* sp. NJUST18 can degrade pyridine. Almost 50 times more melamine can be degraded by this strain of *Micrococcus* sp. than by the strain MF-1. Other degrading bacteria, single degradable pollutants, low degrading efficacy of refractory pollutants, and difficult degrading conditions are disadvantages. *Nocardioides* has the advantage of dealing with a wide range of pollutants, including those from medicine, industry, materials, and many other fields. Nitrogen heterocyclic compounds can completely degrade refractory pollutants such as plastics, the conditions for degradation are broad and easy to implement, the degradation time is short, and the degradation efficiency is high. *Nocardioides* is expected to provide materials for environmental bioremediation because of this uniqueness. 

*Nocardioides* also has other unique applications. *Nocardioides* can resist metal [107], remove toxins, and affect blooms. For example, Li et al. isolated *Nocardioides* sp. from Hg-contaminated soil [108]. In Hg-contaminated soil, *Nocardioides* sp. is the dominant flora and can be used as a biological indicator of metal pollution [109]. Additionally, Bagade et al. isolated *Nocardioides* sp. L-37a [110] from an arsenic (As)-contaminated environment with arsenate reductase activity. This indicates that *Nocardioides* sp. also has significant application potential in the degradation of the carcinogen As and its compounds. YokoIkunaga found that *Nocardioides* sp. strain WSN05-2 was able to eliminate 1000 μg/L of emetic toxin (DON) within 10 d [43]. *Nocardioides lacusdianchii* sp., which can promote *Microcystis aeruginosa* growth and induce the formation of a *Microcystis aeruginosa* population, was isolated by Xiao et al. from a *Microcystis aeruginosa* culture [111]. Additionally, it is essential for the emergence, spread, and reduction of microcystis bloom. In conclusion, *Nocardioides* offers an excellent research space, and their application prospects in the agricultural, industrial, and pharmaceutical industries are inestimable.

*Nocardioides* has good contaminant degradation capacity and can biodegrade and catalyze steroids. Their current bioprocessing mainly focuses on microbial degradation and biotransformation catalysis. In terms of biotransformation, *Nocardioides* has a variety of biocatalytic enzymes. For example, *Nocardioides* sp. YR527 can produce vanillin on a large scale using eugenol oxidase [112]. In terms of pollutant degradation, *Nocardioides* often forms complex bacteria with other microorganisms [113]. For example, a *Nocardioides* complex can produce biosurfactants that dissolve petroleum hydrocarbons and facilitate microbial utilization [114]. In terms of commercial applications, it is expected that *Nocardioides* will be used to develop microbial agents with application value. In addition, their multiple biocatalytic enzymes can degrade and bioconvert steroids; this opens up new perspectives for the steroid pharmaceutical industry to create effective biocatalysts.

However, with the advancement of bioinformatics, the methods of whole-genome sequencing, genome assembly, and gene function prediction are gradually maturing. This is due to the late start of research on this strain which causes the degradation of environmental pollutants to still evolve. Gene function prediction analysis can be integrated with the gene information of *Nocardioides* and the functional genes enriched in a particular environment to confirm the functional genes. Therefore, it is increasingly important to study the structure and biological functions of *Nocardioides*. Simultaneously, *Nocardioides* is expected to develop into a microbial agent with significant market and application value based on existing strains’ excellent pollutant degradation ability. Humans are expected to find new, more valuable *Nocardioides* species and more biological functions soon.

## Figures and Tables

**Table 1:** Types of *Nocardioides* degradation contaminants and their degradability.

Pollutant Type	Strain Name	Degradation Efficiency and Initial Concentration	Degradation Time	Strain Source	Medium Type	Culture Conditions	References
Nitrophenol	*Nocardioides* sp. KP7	100% -	24 h	Marine	BSM medium	30 °C PH 7	[33]
	*Nocardioides nitrophenolicus* sp. NSP41T	- Initial conc.: 200 mg/L	-	Industrial wastewater	Difo medium	30 °C PH 7	[34]
Dibenzofuran	*Nocardioides aromaticivorans*	100% 33 mg/L	96 h	Surface water	PBY medium	30 °C PH 7	[26]
2,4,6-Trinitrophenol (picric acid)	*Nocardioides simplex* FJ2-1A	78% Initial conc.: 146 mg/L	28 d	Picric acid-containing wastewater	BSV medium	30 °C PH 7.4	[30]
2,4-Dinitrophenol	*Nocardioides* sp. JS1661	100% Initial conc.: 150 mg/L	45 h	Soil	MSB medium	30 °C PH 6.5	[29]
Ibuprofen	*Nocardioides carbamazepini* sp. nov.	70% Initial conc.: 1.6 mg/L	Seven weeks	Groundwater	R2A medium	28 °C PH 7	[32]
Propoxur	*Nocardioides* sp. SP1b	100% Initial conc.: 100 mg/L	60 h	Soil	PTYG medium	28 °C PH 7	[35]
Pyridine	*Nocardioides* sp. strain OS4	100% 5 g/L	Two weeks	Oxic zone of a spent shale column	R2A medium	28 °C PH 7	[36]
Ritalinic acid	*Nocardioides* sp. strain MW5	100% Initial conc.: 1 g/L	4 h	Arsenic springs	Mineral medium + ritalinic acid	30 °C PH 7	[37]
Atrazine	*Nocardioides* sp. strain DN36	100% 0.9 mg/L	7 d	Soil	R2A medium	30 °C PH 7	[38]
Cotinine	*Nocardioides* sp. strain JQ2195	100% Initial conc.: 500 mg/L	30 h	Wastewater	MSM medium + cotinine	30 °C PH 7	[27]
Melamine	*Nocardioides* sp.	100% Initial conc.: 5.04 g/L	20 d	Soil	LMM medium	30 °C PH 7	[39]
Polylactic acid	*Nocardioides zeae* EA12	2.82% Initial conc.: 6.9 mg/L	35 d	Plastics	TSB medium	30 °C PH 7	[40]
Poly-3-hydroxybutyrate	*Nocardioides. marinisabuli* strain OK12	100% Initial conc.: 318 ± 75 μg/cm^2^	10 d	Plastic film	R2A medium	30 °C PH 7	[41]
Crude oil	*Nocardioides oleivorans* sp. nov.	40% Initial conc.: 50 mg/mL	3 weeks	Crude oil	MSM medium + crude oil	30 °C PH 7	[42]
Vomitoxin (DON)	Nocardioides sp. strain WSN05-2	100% Initial conc.: 1 mg/L	10 d	Soil	Mineral medium + vomitoxin	30 °C PH 7	[43]

Initial conc., initial concentration; d, day; h, hour; -, no data yet.

**Table 2:** Comparison of pollutant degradation capacity of *Nocardioides* sp. with other strains.

Pollutant Type	The Degradability of *Nocardioides* sp.	Other Degrading Bacteria and Degradation Ability	References
Poly-3-hydroxybutyrate	100% degradation of 318 ± 75 μg/cm^2^	*Shewanella* sp. (100% degradation of 47 μg/cm^2^)	[41]
Dibenzofuran	100% degradation of 33 mg/L in 96 h	*Pseudomonas* sp. strain ISTDF1 (40% degradation of 200 mg/L in 36 h)	[102]
		*Pseudomonas aeruginosa* FA-HZ1 (100% degradation of 20 mg/L in 70 h)	[103]
		*Pseudomonas sp.* strain C3211 (100% degradation of 0.585 mg/L in 67 h)	[90]
2,4-Dinitrophenol	100% degradation of 150 mg/L in 45 h	*Rhodococcus erythropolis* strain HL 24-1 and *Rhodococcus erythropolis* strain HL 24-2 (100% degradation of 92 mg/L in 25 h)	[75]
		*Burkholderia* sp. strain KU-46 (100% degradation of 92 mg/L in 6 h)	[104]
Pyridine	100% degradation of 5 g/L in two weeks	*Rhizobium* sp. NJUST18 (100% degradation of 2600 mg/L)	[105]
		*Paracoccus* sp. NJUST30 (100% degradation of 500 mg/L in 54 h)	[106]
Melamine	100% degradation of 5.04 g/L in 20 d	*Micrococcus* sp. strain MF-1 (100% degradation of 100 mg/L in 35 h)	[98]

Refer to Table 1 for the strains of *Nocardioides* that may degrade the above pollutants; d, day.

## Data Availability

Not applicable.
